# Morphology and classification of the second mesiobuccal canal in maxillary first molars: a cone-beam computed tomography analysis in a Chinese population

**DOI:** 10.1186/s12903-024-04363-x

**Published:** 2024-05-14

**Authors:** Yan Xiang, Zhaojun Wu, Lvli Yang, Wei Zhang, Na Cao, Xiaoman Xu, Yao Lin

**Affiliations:** 1 Endodontics Department of Stomatological Hospital of Xiamen Medical College, Xiamen, 361008 China; 2 Xiamen Key Laboratory of Stomatological Disease Diagnosis and Treatment, Huli District, No. 1309, Lvling Road, Xiamen, 361008 Fujian China; 3 Departments of Dental and Maxillofacial Radiology, Stomatological Hospital of Xiamen Medical College, Xiamen, 361008 China

**Keywords:** Cone-beam computed tomography, Maxillary first molar, Root canal configuration, The second mesiobuccal canal, Vertucci’s classification

## Abstract

**Background:**

Understanding the tooth anatomy is crucial for ensuring effective endodontic treatment. This study investigated the root canal morphology of the second mesiobuccal (MB2) canal in maxillary first molars (MFMs) in a Chinese population using cone-beam computed tomography (CBCT).

**Methods:**

This study evaluated 486 MFMs with MB2 canals from 285 participants undergoing CBCT examination and determined the Vertucci’s classification and position of the MB2 canal orifice. The prevalence of the MB2 canal was correlated with the sex, age, and tooth side. The correlations between the prevalence of the MB2 canal and sex and tooth side were assessed using the Fisher's exact test. The chi-square test was used for evaluating the correlation between the prevalence of the MB2 canal and age.

**Results:**

The number of type II, III, IV, V, VI, VII, and other root canals in the MFMs was 30.9%, 0.6%, 65.0%, 1.2%, 1.2%, 0.4%, and 0.6%, respectively. Among the 201 cases with bilateral inclusion, 87.6% showed consistent canal configuration. Results of the first clear apparent position (FCAP) of the MB2 canals showed that 434, 44, and 3 teeth had FCAP at the upper, middle, and bottom one-third of the root, respectively. The FCAPs of the MB2 canal in the MFMs with types II, IV, and VI, as well as types III and V canals showed significant differences (*p*<0.05). The horizontal distance between the MB1 and MB2 canal orifices in the type II canals of MFMs was significantly lesser than those in the type IV canals of MFMs (*p* < 0.01). The longitudinal distance between the pulp chamber floor plane and MB2 canal orifice significantly correlated with age (*p* < 0.05).

**Conclusions:**

The morphology of the mesiobuccal root canal in the MFMs is complex. Complete understanding of the anatomical morphology of the root canal combined with the CBCT and dental operating microscope is necessary for the accurate detection of the MB2 canal and consequently improved success rate of root canal treatment. Our study findings can help endodontists improve endodontic treatment outcomes.

**Supplementary Information:**

The online version contains supplementary material available at 10.1186/s12903-024-04363-x.

## Background

The goal of root canal treatment is the thorough cleaning and shaping of the root canal to allow complete obturation with inert filling materials to eliminate necrotic tissue and prevent the penetration of microorganisms into the monoblock system of the root canal [1, 2]. A better understanding of the root canal morphology and its variations is crucial for successful endodontic treatment [1]. The prevalence of a second mesiobuccal (MB2) canal in the maxillary molars is reported to be over 50% [3–6]. Race, age, and sex cause wide variations in the prevalence of the MB2 canal [1]. Complete understanding of the complex anatomy of the root canal is necessary for proper management as accurate diagnosis contributes to successful root canal treatment [2]. The MB2 canals in the maxillary first molars (MFMs) have small and curved calcified root canals, difficult to detect root canal orifices, and complex root canal types, which are prone to undetected root canals and failure in root canal treatment in clinical practice. Therefore, the presence of the MB2 canal should be considered before treatment [1].

Various methods, such as the canal staining and clearing technique, spiral computed tomography (CT) thin-layer scanning, micro-CT, radiographic examination, cone-beam CT (CBCT), and dental operating microscope, have been employed for evaluating the root canal morphology [3, 7–11]. CBCT has been used to assess the root canal morphology owing to its short exposure time, low cost compared to that of conventional CT, high resolution and accuracy, minimal distortion, three-dimensional visualization, and noninvasiveness [1, 5, 6, 12]. This study employed CBCT and its three-dimensional reconstruction data to study the classification of the MB2 canal in the MFMs, location of the MB2 canal, horizontal distance between the MB1 and MB2 canal orifices (HD), and longitudinal distance between the MB2 canal orifices and pulp chamber floor plane (LD) in a Chinese population to provide guidance for clinical root canal treatment.

## Methods

### Study participants

This study was approved by the Ethics Committee of Stomatological Hospital of Xiamen Medical College (IRB No. EC-20221025-1029). Two-hundred and eighty-five patients with MB2 canals in their MFMs requiring CBCT examination as part of their dental diagnosis and treatment underwent CBCT scanning at the Stomatological Hospital of Xiamen Medical College between January 2020 and September 2021. The patients consisted of 117 men and 168 women with an age range of 18–74 years; 486 MFMs (241 right and 245 left MFMs) were evaluated.

### Inclusion and exclusion criteria

Inclusion criteria were teeth with complete pulp cavity of the MFM, complete root development, no resorption, no longitudinal cracks, clear imaging, and complete clinical data. Exclusion criteria were teeth with artifacts caused by root canal obturation, post and crown restoration, destruction of the pulp cavity integrity, severe calcification of the pulp cavity or root canal, or incomplete development of the tooth roots, which could affect the interpretation of the root canal morphology.

### CBCT analysis of the MB2 canal in the MFMs

CBCT was conducted using a NewTom VGi CBCT device (QRsrl, Verona, Italy) with 110 kV of voltage, 5.49 mA of galvanic current, and 5.4-s exposure time. The scan resolution was 125×125×125 μm. The display screen was 30.4-inch MDCC-6430 coronis fusion 6MP LED (Barco, Poperinger, Belgium) with a resolution of 3280×2048 pixels.

Multiplannar reformation (MPR) function of the NNT software (NewTom,Verona, Italy) was used for the data analysis. The horizontal, sagittal, and coronal images of the MFMs were observed by continuously moving the roller from the bottom of the pulp chamber to the apical area in the image analysis software after opening the CBCT images; the position of the MB2 canal orifice, classification and bilateral symmetry, horizontal distance between the MB1 and MB2 canal orifices (HD), and longitudinal distance between the MB2 canal orifices and pulp chamber floor plane (LD) were measured. All the CBCT images were evaluated by an experienced oral and maxillofacial imaging physician and a dental and endodontic physician separately, and the reliability of their results were tested using the kappa test. Inconsistency in the results between the two physicians was resolved by a third senior imaging physician.

The MB2 canals in the MFMs were classified using Vertucci classification, which is based on the anatomical morphology of the main root canal of the tooth root (Additional file 1) [1].

The highest point of the pulp chamber floor serves as the positioning measurement plane, i.e., the root canal orifice. The contour of the root canal orifice was determined based on the grayscale threshold. The position of the root canal orifice is defined by the mass center coordinate of the root canal orifice contour. The HD was measured (Additional file 1). The second observation plane was the first clear apparent position (FCAP) of the MB2 canal from the positioning measurement plane to the root tip direction, and the LD was measured (Additional file 1). Simultaneously, the FCAP of the MB2 canal in the MFMs in the CBCT images was also analyzed and divided into the top 1/3, middle 1/3, and bottom 1/3 groups (Additional file 1).

### Data analysis

The correlations between the prevalence of the MB2 canal and sex and tooth side were assessed using the Fisher's exact test. The chi-square test was used for evaluating the correlation between the prevalence of the MB2 canal and age. Pearson correlation analysis was conducted using the R basicTrendline package. All the analyses were completed using R 4.2.3. P < 0.05 was considered statistically significant.

## Results

The inter-examiner reliability was 0.827.

### Classification of the MB2 canals in the MFMs

The number of roots in each of the 486 MFMs from 285 patients (190 MBs from 117 men, and 296 MBs from 168 women) was assessed (Table 1). The number of type II, III, IV, V, VI, VII, and other canals in the MFMs was 150 (30.9%), 3 (0.6%), 316 (65.0%), 6 (1.2%), 6 (1.2%), 2 (0.4%), and 3 (0.6%), respectively (Table 1) (Additional file 2). Among the 201 cases with bilateral inclusion, 176 (87.6%) had consistent root canal classification in the MFMs. Furthermore, no significant difference was observed in the Vertucci classification of the MB2 canal in the MFMs between men and women (Fisher’s exact test for count data, *p* = 0.6641).

**Table 1:** Vertucci classification of the second mesiobuccal canals in maxillary first molars

Vertucci Classification	Man	Woman	Total
II	54 (28.42%)	96 (32.43%)	150 (30.9%)
III	1 (0.53%)	2 (0.68%)	3 (0.6%)
IV	127 (66.84%)	189 (63.85%)	316 (65.0%)
V	3 (1.58%)	3 (1.01%)	6 (1.2%)
VI	4 (2.11%)	2 (0.68%)	6 (1.2%)
VII	0 (0%)	2 (0.68%)	2 (0.4%)
Other	1 (0.53%)	2 (0.68%)	3 (0.6%)
Total	190 (100%)	296 (100%)	486 (100%)

### The FCAP of the MB2 canal in the CBCT images

**Fig. 1:**
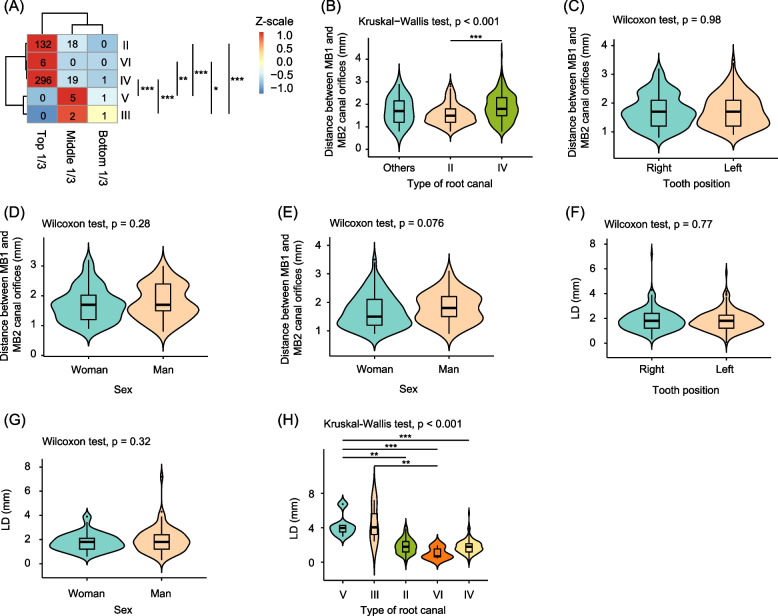
Position of the second mesiobuccal (MB2) canal in the maxillary first molars (MFMs). **A** position of the MB2 canal in the MFMs with clear image in cone-beam computed tomography analysis. **B**-**E** Differences in the horizontal distance between the MB1 and MB2 canal orifices in the MFMs. **B** Type of MB2 canal. **C** Tooth position. **D** Difference between sexes in the right MFMs. **E** Difference between sexes in the left MFMs. **F**-**G** The longitudinal distance between the MB2 canals and chamber floor between different dental positions and between different sexes. **H** The longitudinal distance between the MB2 canal orifices and chamber floor in the different root canal types. * *p* < 0.05; ** *p* < 0.01; *** *p* < 0.001

Considering the results of the location of the MB2 canal in the 481 MFMs with a distribution of root canal types > 1% showed that the MB2 canal of 434, 44, and 3 teeth was located in the top, middle, and bottom 1/3 of the canal, respectively. Significant differences were observed between the FCAP of the MB2 canal in the MFMs with types II, IV, and VI, as well as types III and V (Fisher's exact test, *p* < 0.05; Fig. 1A). These findings indicated that different root canal types in the MFMs had different FCAP in CBCT. The FCAP of the MB2 canal of types II, IV, and VI were mainly located in the top 1/3 of the teeth, while those of the MB2 canal of types IIIII and V were mainly located in the middle 1/3 of the teeth (Fig. 1A). Among the 201 bilateral MFMs included in the study, the FCAP of the MB2 canal in the MFMs was consistent in 181 cases (90.0%).

### HD and LD of the MFMs

**Fig. 2:**
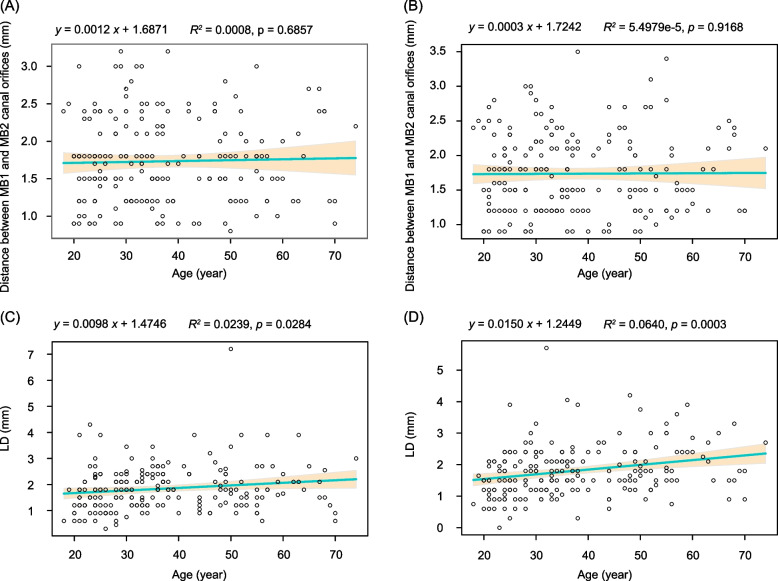
Correlations between the positions of the second mesiobuccal (MB2) canals and age. **A** right maxillary first molar (MFM); (**B**) left MFM; (**C**) right MFM; (**D**) left MFM. Distance between the first mesiobuccal (MB1) and MB2 canal orifices is transverse distance. LD is the longitudinal distance between the chamber floor plane and MB2 canal orifices

The HD in type II canals of the MFMs was significantly lesser than that in the type IV canals of the MFMs (Wilcoxon test, *p* < 0.01; Fig. 1B). However, the HDs between the right and left MFMs did not show significant difference (Wilcoxon test, *p* = 0.98; Fig. 1C). No significant difference was observed in the HDs between the sexes in the right (Wilcoxon test, *p* = 0.28, Fig. 1D) or left MFMs (Wilcoxon test, *p* = 0.078, Fig. 1E). No significant difference was observed in the LD between the different tooth positions (Wilcoxon test, *p* = 0.77; Fig. 1F) and sexes (Wilcoxon test, *p* = 0.32; Fig. 1G). The LD of types II, IV, and VI MFMs was significantly lower than that of type V MFMs (Kruskal–Wallis test, *p* < 0.001; Fig. 1H), and that of type VI MFMs was significantly lower than that of type III MFMs (Kruskal–Wallis test, *p* < 0.01; Fig. 1H). Furthermore, the LD significantly correlated with age (Pearson correlation analysis, *p* < 0.05; Fig. 2C and 2D), whereas the HD did not significantly correlate with the age (Pearson correlation analysis, *p* > 0.05; Fig. 2A and B).

## Discussion

Dental anatomy variations are reportedly influenced by several factors, namely ethnicity, age, and sex. The maxillary first premolars are the most difficult teeth to be treated. Factors contributing to the challenging treatment include the number of roots, number of root canals, direction of the root canal, configuration of the pulp cavity, and difficulty in visualizing the apical dimension. Many previous studies have demonstrated the association of the morphological variations and number of root canals in the maxillary first premolars with problems in root canal treatment [13].

CBCT is widely used in the clinical evaluation of tooth roots and root canal anatomy owing to its high accuracy, minimal distortion, three-dimensional visualization, and noninvasiveness [1, 14]. The American Association of Endodontists and American Academy of Oral and Maxillofacial Radiology have recommended the use of limited field of view CBCT for teeth with suspicious complex shapes or potential root canals in patients with pulp diseases during initial treatment [15]. The advantage of traditional CT imaging is that CBCT obtains all the data in one scan, resulting in a significant reduction in the scan time and radiation dose. Matherne et al. [16] reported that the use of CBCT imaging resulted in better identification of the root canals upon comparing charge-coupled device and photostimulable phosphor plate digital radiography. In this study, we used CBCT to study and analyze the root canal types through a three-dimensional perspective using the FCAP of the MB2, HD, and LD. Our findings provide clinical guidance to dentists concerning MB2 canal detection, thereby improving the success rate of root canal treatment.

Numerous previous studies have demonstrated that the most common anatomical type of the MFM is three roots with four root canals; the root canal system in the mesiobuccal root of the MFM is complex, with an incidence of MB2 canals exceeding 50% [6]. However, certain differences exist in the incidence among different studies, which may be attributed to the differences in race, age, sex, and study methods. Type IV canals in the MFM mesiobuccal roots are more common in Asians, which is contradictory to the findings of the studies on the White populations in Turkey [17] and the United States [18], where the most common canal configuration was type II. However, other studies reported different results from those found in a Turkish population [19], which determined type IV to be the most common canal configuration; moreover, in the US population [6], a similar prevalence of types II and IV canal configurations was found. Pérez-Heredia et al. [20] reported that the proportions of type II, IV, VI, and other canals in 119 teeth were 65.5%, 26.9%, 5.0%, and 2.5%, respectively. Ratanajirasut et al. [1] reported that the proportions of type II, III, IV, V, VI, and other canals in 302 teeth of a Thai population were 45.4%, 4.3%, 39.7%, 8.3%, 1.7%, and 0.7%, respectively. Mheiri et al. [21] reported that the proportions of type II, III, and IV in 418 teeth were 73.7%, l7.2%, and 19.1%, respectively. Mufadhal and Madfa [22] reported that the proportions of type II, III, IV, V, VI, VII, and others in 306 teeth were 30.4%, 28.1%, 13.7%, 6.9%, 8.2%, 2.6%, and 10.1%, respectively. These differences are probably attributed to the different regions, races, genders, or ages [1, 20–22]. Our results demonstrated that type IV MFMs constituted the highest proportion, accounting for 65.0%, followed by type III MFMs accounting for 30.9%. Other types, such as types III, V, VI, and VII, accounted for approximately 1%. Types IV and II MFMs accounted for more than 90% of the multi canal types in the MFMs. Our study findings were similar to those of Ratanajirasut et al.'s [1] study in a Thai population and Qiao et al.'s [23] and Jin et al.'s [24] studies in other Chinese populations.

Understanding the morphology of the buccal root canal of the MFMs is crucial for improving the success rate of root canal treatment. The analysis of the HDs in the MFMs in our study demonstrated a significant difference between the HDs of types IV and II MB2, indicating that the HDs of the mesiobuccal root of the MFMs varied based on the root canal type, with that of type IV being greater than that of type II. X-ray imaging in combination with knowledge if the root canal classification aids in the location of the MB2 canal orifice in clinical practice. Our findings were similar to those by Srivastava et al. [25] in a Saudi population. Our results showed that in the case of multiple canals in the mesial buccal root canal, the HD of type IV MB2 (3.8±0.21 mm) was significantly greater than that of type II MB2 (2.4±0.47 mm). Furthermore, the FCAP results of MB2 canal in the MFMs indicated that 90% of the cases were located in the upper 1/3 of the tooth. The LD of types II, IV, and VI MFMs was significantly lower than that of type V MFMs, and the LD of type VI MFMs was significantly lower than that of type III MFMs, indicating that different canal types in the MFMs had different FCAP. Commonly, the FCAP of the types II, IV, and VI MB2 was mainly located in the upper 1/3 of the tooth. Therefore, during clinical operations, the ultrasonic working tip under a dental microscope can effectively explore the MB2 canal orifices by removing calcified dentin of approximately 3 mm.

The shape and structure of the tooth root canals change with age. Our study suggested that LD decreases with age possibly due to the gradual narrowing of the pulp cavity and calcification of the root canal [26], which makes the identification and preparation of the MB2 canal during root canal treatment more challenging, thereby increasing the difficulty of exploration. Moreover, our study also found that the LD correlated with the root canal type, with that of type III being significantly higher than that of type VI, and that of type V being significantly higher than those of types II, IV, and VI. The study of the HD and LD provided a basis for clinical localization of the MB2 canal.

The need for root canal retreatment in several molars is associated with missed MB2 canal preparation during initial treatment. This study analyzed the condition of the mesiobuccal root canals of the MFM to provide guidance for understanding the morphology of the root canals. Khalighinejad et al. [27] reported the probability of root canal retreatment occurring without the use of dental microscopes during root canal treatment to be three times higher than that with the use of dental microscopes. More attention should be paid when treating the buccal root of the MFM; identifying the position of the MB2 canal is crucial for the success of root canal treatment.

Although CBCT has the advantages mentioned above, its shortcomings, including a possible higher radiation dose to the patient, and potential for artifact generation, high levels of scatter and noise, and variations in dose distribution within a volume of interest, limit CBCT widespread clinical application [28]. Therefore, exploring the factors that affect morphology and classification of the MB2 canal and establishing a predictive model for morphology and classification of the MB2 canal is of great clinical significance for root canal treatment. However, due to the influence of complex factors on morphology and classification of the MB2 canal, it is difficult to achieve the above goals without a large amount of clinical survey data. This study not only provided clinical data for constructing models for predicting morphology and classification of the MB2 canal, but also revealed the significant changes in LD with age. Although these current results did not provide direct guidance for endodontists, they could still provide some hints about morphology and classification of the MB2 in Chinese populations.

## Conclusions

The morphology of the mesiobuccal root canal in the MFMs is complex making root canal treatment challenging. Complete understanding of the anatomical morphology of the root canal, combined with CBCT and dental operating microscope is necessary for the accurate detection of the MB2 canal, avoiding excessive dental tissue removal during exploration, preserving the dentin of the tooth neck, avoiding complications, such as lateral and bottom penetration, and improving the success rate of root canal treatment. Our results can help endodontists improve endodontic treatment outcomes.

### Supplementary Information

Supplementary Material 1. 

## Data Availability

The datasets used or analyzed during the current study are available from the corresponding author upon reasonable request.

## Abbreviations

MB2: Second mesiobuccal
MFM: Maxillary first molar
CBCT: Cone-beam computed tomography
FCAP: First clear apparent position

## References

[1] Ratanajirasut R, Panichuttra A, Panmekiate S (2018). A cone-beam computed tomographic study of root and canal morphology of maxillary first and second permanent molars in a Thai population. J Endod.

[2] Hernawatiningsih Kristanti Y, Subandhi DH (2021). Root canal treatment on Vertucci type V configuration – A case report. Adv Health Sci Res.

[3] Zheng QH, Wang Y, Zhou XD, Wang Q, Zheng GN, Huang DM (2010). A cone-beam computed tomography study of maxillary first permanent molar root and canal morphology in a Chinese population. J Endod.

[4] Kim Y, Lee SJ, Woo J (2012). Morphology of maxillary first and second molars analyzed by cone-beam computed tomography in a Korean population: variations in the number of roots and canals and the incidence of fusion. J Endod.

[5] Zhang R, Yang H, Yu X, Wang H, Hu T, Dummer PMH (2011). Use of CBCT to identify the morphology of maxillary permanent molar teeth in a Chinese subpopulation. Int Endod J.

[6] Guo J, Vahidnia A, Sedghizadeh P, Enciso R (2014). Evaluation of root and canal morphology of maxillary permanent first molars in a North American population by cone-beam computed tomography. J Endod.

[7] Alavi AM, Opasanon A, Ng YL, Gulabivala K (2002). Root and canal morphology of Thai maxillary molars. Int Endod J.

[8] Somma F, Leoni D, Plotino G, Grande NM, Plasschaert A (2009). Root canal morphology of the mesiobuccal root of maxillary first molars: a micro-computed tomographic analysis. Int Endod J.

[9] Wen S, Lin Z, Zhu M, Ge J, Wang T (2016). Comparative study of root canal morphology of mandibular incisors by cone-beam CT and canal staining and clearing technique. Prog Geog.

[10] Society of Cariology and Endodontics, Chinese Stomatological Association. Guidelines for radiographic examination in cariology and endodontics. Chin J Stomatol. 2021;56(4):311–7. 10.3760/cma.j.cn112144-20210125-00039. 10.3760/cma.j.cn112144-20210125-00039 33832030

[11] Liang A, Huang L, Li B, Huang Y, Zhou X, Zhang X, Gong Q (2022). Micro-CT evaluation of different root canal irrigation protocols on the removal of accumulated hard tissue debris: a systematic review and meta-analysis. J Clin Med.

[12] Lin YH, Lin HN, Chen CC, Chen MS (2017). Evaluation of the root and canal systems of maxillary molars in Taiwanese patients: a cone beam computed tomography study. Biomed J.

[13] Awawdeh L, Abduallah H, Al-Qudah A (2008). Root form and canal morphology of Jordanian maxillary first premolars. J Endod.

[14] Ren HY, Kum KY, Zhao YS, Yoo YJ, Jeong JS, Perinpanayagam H, Wang XY, Li GJ, Wang F, Fang H, Gu Y (2021). Maxillary molar root and canal morphology of Neolithic and modern Chinese. Arch Oral Biol.

[15] Special Committee to Revise the Joint AAE/AAOMR Position Statement on use of CBCT in Endodontics. AAE and AAOMR Joint Position Statement. Use of cone beam computed tomography in endodontics 2015 update. J Endod. 2015;41(9):1393–6. 10.1016/j.joen.2015.07.013. 10.1016/j.joen.2015.07.013 26320105

[16] Matherne RP, Angelopoulos C, Kulild JC, Tira D (2008). Use of cone-beam computed tomography to identify root canal systems in vitro. J Endod.

[17] Çalişkan MK, Pehlivan Y, Sepetçioglu F (1995). Root canal morphology of human permanent teeth in a Turkish population. J Endod.

[18] Plotino G, Tocci L, Grande NM (2013). Symmetry of root and root canal morphology of maxillary and mandibular molars in a white population: a cone-beam computed tomography study in vivo. J Endod.

[19] Altunsoy M, Ok E, Nur BG (2014). A cone-beam computed tomography study of the root canal morphology of anterior teeth in a Turkish population. Eur J Dent.

[20] Pérez-Heredia M, Ferrer-Luque CM, Bravo M, Castelo-Baz P, Ruíz-Piñón M, Baca P (2017). Cone-beam computed tomographic study of root anatomy and canal configuration of molars in a Spanish population. J Endod.

[21] Mheiri EAI, Chaudhry J, Abdo S, Abed REI, Khamis AH, Jamal M (2020). Evaluation of root and canal morphology of maxillary permanent first molars in an Emirati population; a cone-beam computed tomography study. BMC Oral Health.

[22] Mufadhal AA, Madfa AA (2023). The morphology of permanent maxillary first molars evaluated by cone-beam computed tomography among a Yemeni population. BMC Oral Health.

[23] Qiao X, Xu T, Chen L, Yang D. Analysis of root canal curvature and root canal morphology of maxillary posterior teeth in Guizhou, China. Med Sci Monit. 2021;27:e928758. 10.12659/MSM.928758. 10.12659/MSM.928758 PMC7812699 33439855

[24] Jin Y, Yuan L, Zhou Y, Huang X, Xiao X (2014). Study of root and canal morphology of maxillary first and second molars evaluated by cone beam computed tomography. J Oral Sci Res.

[25] Srivastava S, Aldakhail NS, Javed MQ (2023). Morphometric relationships in mesiobuccal roots of maxillary first molars in Saudi subpopulation: A CBCT study. Aust Endod J.

[26] Sue M, Oda T, Sasaki Y, Ogura I (2018). Age-related changes in the pulp chamber of maxillary and mandibular molars on cone-beam computed tomography images. Oral Radiol.

[27] Khalighinejad N, Aminoshariae A, Kulild JC, Williams KA, Wang J, Mickel A (2017). The effect of the dental operating microscope on the outcome of nonsurgical root canal treatment: a retrospective case-control study. J Endod.

[28] AAE and AAOMR Joint Position Statement (2015). Use of cone beam computed tomography in endodontics 2015 update. J Endod.

