# Supplementary material for: Morphology and classification of the second mesiobuccal canal in maxillary first molars: a cone-beam computed tomography analysis in a Chinese population
Source: BMC Oral Health. 2024 May 14;24:568. doi: 10.1186/s12903-024-04363-x (PMC11094936; doi:10.1186/s12903-024-04363-x)

**Additional file 1**. Schematic representation of the second mesiobuccal canal Vertucci classification in maxillary first molars (A), and measurement example of LD (B-G) and HD (H-I) in this study. (B), (D), and (F) were axial images, and (C), (E), and (G) were sagittal images. (B) and (C) Bottom plane of pulp chamber indicated by blue line in sagittal position; (D) and (E) the first clear appear positions of MB2 canals indicated by blue line in sagittal position; (F) and (G) apical plane indicated by blue line in sagittal position. HD, the horizontal distance between MB1 and MB2 canal orifices; LD, the longitudinal distance between MB2 canal orifices and pulp chamber floor plane.


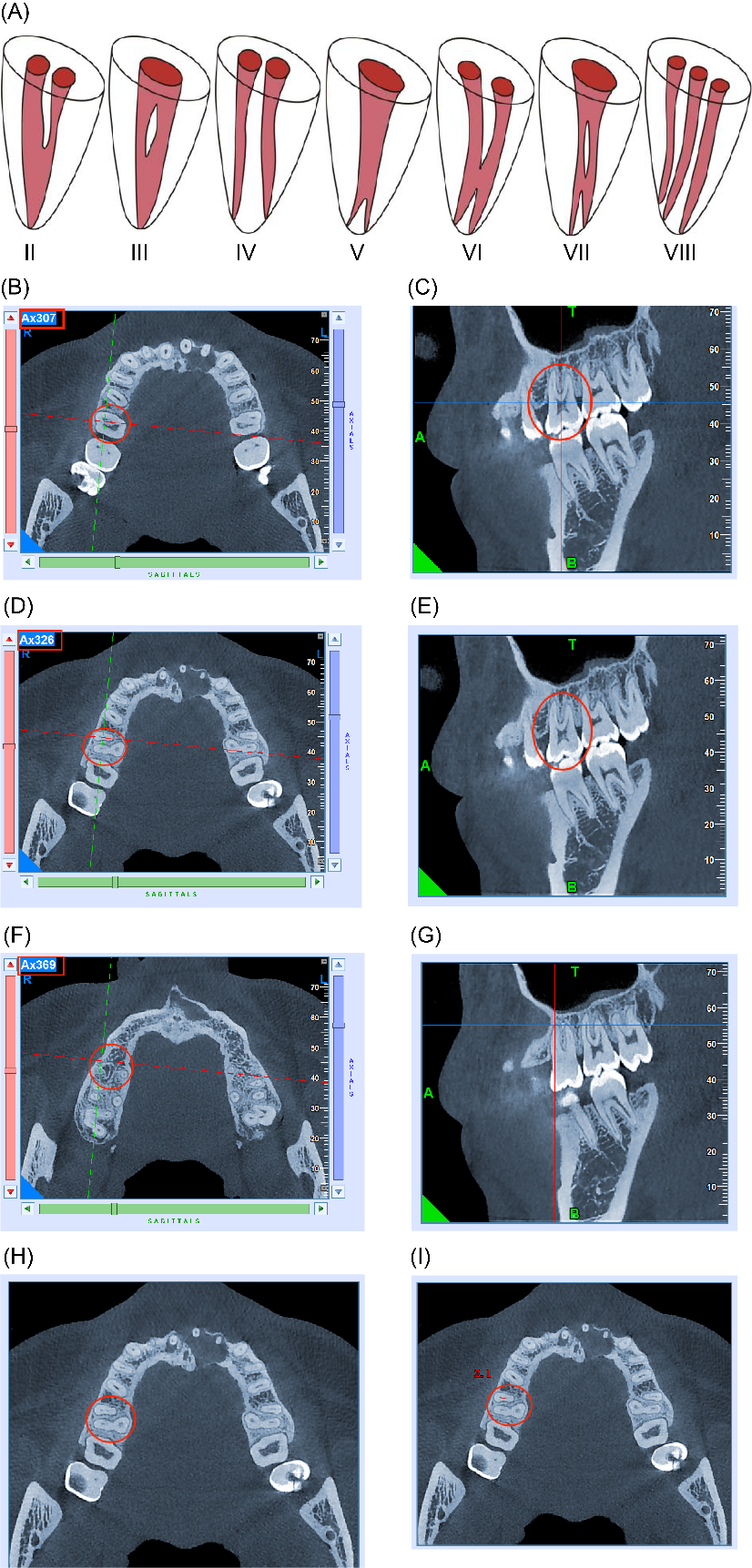


**Additional file 2**. Maxillary first molars. The root canal classification of the MB2 canal in the MFMs was based on the Vertucci classification.


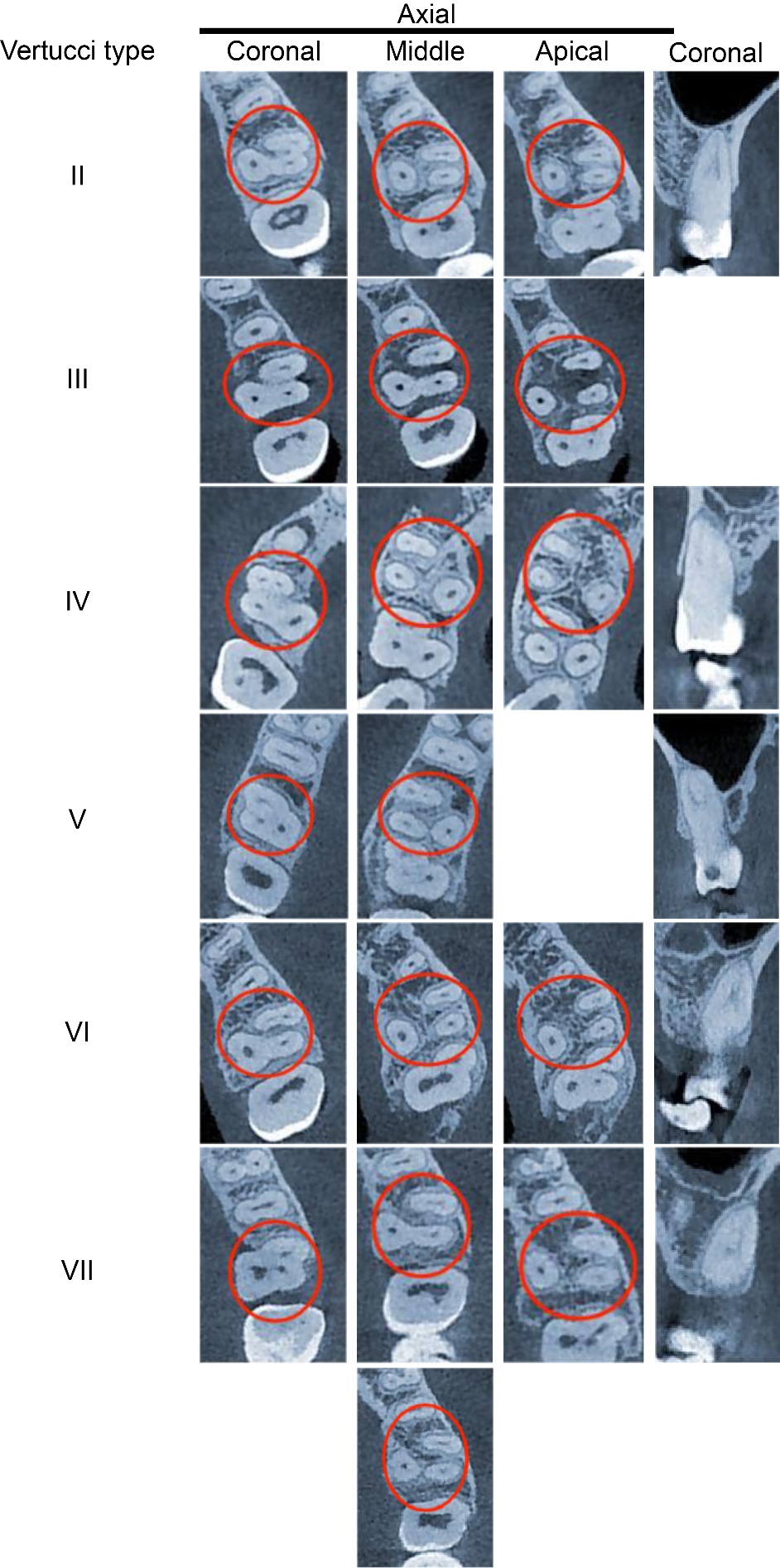

Supplement: Supplementary file 1 — Supplementary Material 1. [file 12903_2024_4363_MOESM1_ESM.docx]
